# High spatial resolution prediction of tritium (^3^H) in contemporary global precipitation

**DOI:** 10.1038/s41598-022-14227-5

**Published:** 2022-06-17

**Authors:** Stefan Terzer-Wassmuth, Luis J. Araguás-Araguás, Lorenzo Copia, Leonard I. Wassenaar

**Affiliations:** Isotope Hydrology Section, International Atomic Energy Agency, Vienna International Centre, 1400 Vienna, Austria

**Keywords:** Hydrology, Environmental monitoring

## Abstract

Tritium (^3^H) in Earth’s precipitation is vigilantly monitored since historical nuclear bomb tests because of radiological protection considerations and its invaluable role as a tracer of the global water cycle in quantifying surface, groundwater, and oceanic fluxes. For hydrological applications, accurate knowledge of ^3^H in contemporary local precipitation is prerequisite for dating of critical zone water and calibrating hydrogeologic transport and groundwater protection models. However, local tritium input in precipitation is hard to constrain due to few ^3^H observation sites. We present new high-spatial resolution global prediction maps of multi-year mean ^3^H in contemporary “post-bomb” (2008–2018) precipitation by using a robust regression model based on environmental and geospatial covariates. The model accurately predicted the mean annual ^3^H in precipitation, which allowed us to produce global ^3^H input maps for applications in hydrological and climate modelling. The spatial patterns revealed natural ^3^H in contemporary precipitation sufficient for practical hydrological applications (1–25 TU) but variable across continental regions and higher latitudes due to cumulative influences of cyclical neutron fluxes, stratospheric inputs, and distance from tropospheric moisture sources. The new ^3^H maps provide a foundational resource for improved calibration of groundwater flow models and critical zone vulnerability assessment and provides an operational baseline for quantifying the potential impact of future anthropogenic nuclear activities and hydroclimatic changes.

## Introduction

Tritium (^3^H; half-life 4500 ± 8 days) is a hydrogen radioisotope widely used to study water movement in terrestrial critical zones, the oceans, and to estimate the residence times of water in near-surface aquifers and surface water bodies^1–6^. Tritium in environmental water is often expressed in TU (Tritium Units), which equals to one molecule of ^3^H^1^HO per 10^18^ molecules of ^1^H_2_O, or 0.11919 Bq kg^−1^^7^, and is produced naturally in ultra-trace quantities in Earth’s upper atmosphere via by the interaction of cosmic rays with N nuclei, at significantly higher rates at high geomagnetic latitudes^8^. Tritium quickly oxidizes to form ^1^H^3^HO (water) and thereby enters the global water cycle as water vapour and precipitation.

From 1945 to 1963 ca. 450 surface atmospheric nuclear bomb tests were conducted, thereby injecting large amounts of anthropogenic ^3^H into the stratosphere, increasing by orders of magnitude the natural ^3^H levels in precipitation^9^. Tritium activities on the order of 5000 to 10,000 TU were measured in rainfall in the early 1960s in the Northern hemisphere, challenging human health exposure guidelines for this radioisotope (900–90,000 TU) ^10^. Following the 1963 Partial Nuclear Test Ban Treaty on atmospheric thermonuclear tests, ^3^H concentrations in rain rapidly dropped, followed by a slow return to pre-bomb levels (1–25 TU) by the mid 2000s^11^. Currently, anthropogenic tritium in Earth’s environmental waters is mainly detectable around local sources like nuclear power or fuel reprocessing plants, medical, and other waste-related activities^10^. Historically, there are no direct measurements of pre-nuclear age ^3^H in precipitation before the 1950s; however, estimates for the temperate and cold climates have been made in the 1960’s using pre-1945 vintage wines^12^ and dated ice cores.

Since the 1960s, the International Atomic Energy Agency (IAEA), in cooperation with the World Meteorological Organization, coordinated collection of ^3^H data on the global atmospheric fallout in precipitation through its Global Network of Isotopes in Precipitation (GNIP; https://nucleus.iaea.org/wiser). While the decadal decline in ^3^H in rainfall to pre-nuclear levels (< 1–5 TU for most tropical and subtropical regions) constitutes an analytical detection limit challenge, tritium remains an invaluable short-lived radioisotope for gaining key information on modern freshwater dynamics, critical zone hydrology, and recharge rates to phreatic aquifers. Rising interest in using tritium for protecting Earth’s critical zone water resources necessitates accurate estimates of water replenishment rates powerfully achieved using ^3^H or ^3^H/^3^He groundwater age dating methods^13^ or for the mapping of groundwater vulnerability to pollution^14^. Local and regional hydrological flow models using ^3^H require accurate data on the contemporary tritium content in precipitation. Unfortunately, ^3^H monitoring sites are few and far between, resulting in scientists relying on data from a distal GNIP station or on spatiotemporal extrapolation, which results in best-guess or inaccurate estimates of ^3^H inputs for groundwater modelling, recharge rates, or water flux determinations^15^. Predictive maps alleviate this problem through data-driven models that guide the interpolation of the continuous space between observed ^3^H data points^16^.

## ^3^H in global precipitation

**Figure 1:**
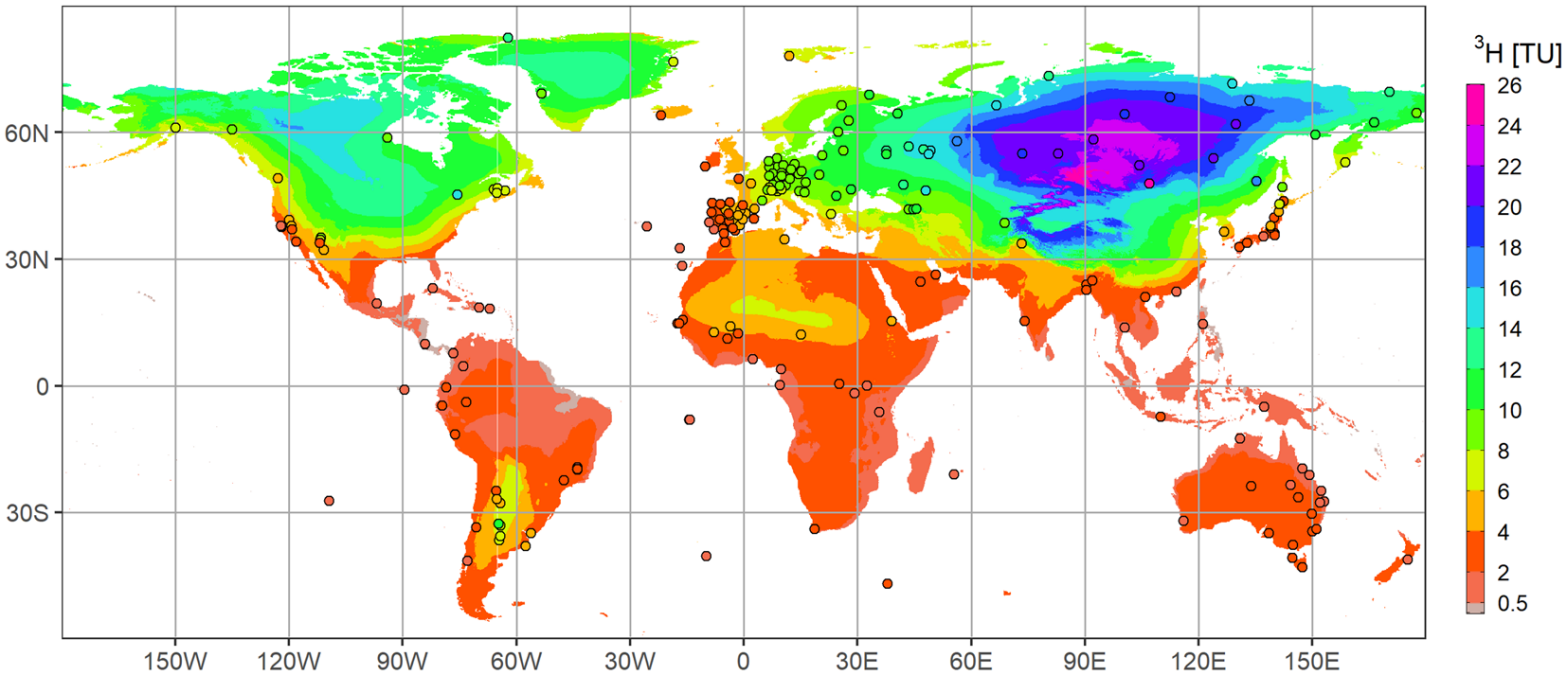
Global patterns of ^3^H in precipitation. Model gridded and measured contemporary (2008–2018) ^3^H contents in Earth’s precipitation at GNIP stations (circles). The tritium distribution map (“isoscape”) was developed using geostatistical techniques described in the Regionalized Cluster-Based Water Isotope Prediction model (RCWIP2). See text and SM1 for details. Figure created in R 4.1.0 (with ggplot2 3.3.5, raster 3.4–13 and rgdal 1.5–23 libraries, all https://cran.r-project.org/).

We examined contemporary global distribution patterns of ^3^H in Earth’s precipitation within the “post-bomb” hydrologic timeframe^11^, in the absence of new anthropogenic ^3^H alterations having global impacts other than natural solar flux cycles^17–19^ and constraining our analysis to the years 2008–18 for data availability reasons. We focused our efforts on monthly precipitation composites and ^3^H data from the IAEA GNIP database^20^ and published data (see discussion in Supplementary Material SM1), with the aim to produce high spatial resolution predictive maps of annual ^3^H in Earth’s rainfall. The curated ^3^H dataset comprised 233 stations for the post-bomb timeframe, with the criteria that they were not affected by anthropogenic sources; most had a minimum continuous monthly record of at least 3 years between 2008 and 2018 (Fig. 1). From this curated dataset, we excluded GNIP sites in Antarctica (n = 4) due to scarcity of observations and the special effects of stratospheric-tropospheric exchange processes controlling ^3^H levels in snow on this continent^21^. We modified IAEA’s regionalized cluster-based water isotope prediction model for stable isotopes in precipitation (RCWIP)^22,23^ by incorporating ^1^H^3^HO as the predictor variable and considering a wide range of explanatory spatial regressors (longitude [LON], weighted latitude [wLT], altitude [ALT], land mass fraction [LMF], and distance to coast [DTC]), as well as climatic and environmental covariates (air temperature [AT], precipitation [PP], radiation-related and other predictors). We selected the best-fit regional predictor model and performed model validation by cross checking the predicted versus observed data (“Methods”).

## Prediction models for ^3^H in global precipitation

Three best-fit multiple-regression models were found to accurately predict ^3^H in worldwide annual precipitation. All models had a high degree of confidence and accuracy, whether it was a (i) global, (ii) extratropical, or a (iii) tropical model (Supplementary Material SM1). The most parsimonious multiple regression predictor models were:

1 $${\text{Global}}\,^{{3}} {\text{H }} \,\left( {{\text{TU}}} \right) \, = \, 0.0313{\text{ wLT }} + \, 2.56 \, 10^{{ - {6}}} \,{\text{DTC }} \,{-} \, 0.2077{\text{ AT }} + \,6.2068{\text{ LMF }} + \, 0.0056{\text{ LONG }} - \, 0.0279{\text{ NLR }} + \, { 61}.{3961}$$

which is a first global model resulting in an R^2^ = 0.79 (*p* < 0.01), where air temperature (AT), wLT, LMF, and DTC explained 26%, 24%, 17% and 9%, respectively, of the overall variance (net longwave radiation [NLR] had only a minor share). As expected, these regressors are the well-known temperature and “continentality effects” on ^3^H distributions^9,24^. The significant response of wLT affirmed the need to specifically account for northern and southern hemispheric differences in the distribution of their land masses. However, the global model (Eq. 1) did not perform as well in tropical and lower latitude regions. Accordingly, it was beneficial to develop the secondary extra-tropical and tropical regional models (see Discussion in^23^):

2 $${\text{Extratropical}}\,^{{3}} {\text{H }} \, = \, - 0.2505{\text{ AT }} + \, 8.0153{\text{ LMF }} + \, { 3}.0{4 \,1}0^{{ - {6}}}\, {\text{ DTC }} + \, 0.0053{\text{ LONG }} + \, 0.0176{\text{ wLT}} \,{-} \,0.0011{\text{ ALT }} + \,71.7114$$

yielding an R^2^ = 0.81 (*p* < 0.01), and with AT, LMF, wLT, and DTC explaining 24%, 23%, 19%, and 12% each of the variability (ALT and LONG were of lesser relevance). For the tropical and low-latitude model, a different set of predictor variables were required, owing to lower cosmogenic ^3^H production rates and substantial dilution by low-^3^H tropical oceanic moisture sources:

3 $${\text{Tropical}}\,^{{3}} {\text{H }} \,= \, 0.0513{\text{ wLT }} + \, 2.2882{\text{ LMF }} + \, 0.0061{\text{ AT }}{-} \, 0.0399{\text{ OLR }} \,{-} \, 0.059{\text{ CPN }} + \, 0.0003{\text{ PP }} + \, { 9}.{3437}$$

which yielded an R^2^ = 0.62, p < 0.01) and whose variability was explained 27% by wLT, 15% by LMF, and the remaining variance equally distributed over four climatic parameters (AT, outgoing longwave radiation [OLR], convective precipitation amount [CPN] and PP). Wherever sufficient ^3^H data was available, RCWIP clusters were calculated with the appropriate regionalized regression equations. For clusters with ^3^H data deficiency, the tropical and extra-tropical models were used as replacements for the global model. The resulting prediction models were applied and merged for each 0.1 × 0.1 degree grid cell using fuzzy clustering climatic zone membership fractions^22^ to create a single unified prediction map of contemporary ^3^H in annual global precipitation (Fig. 1).

## Results and prediction model validation

Our predictive models revealed that contemporary ^3^H in precipitation ranged from near to below instrumental detection limits of ca. 0.5 TU to as high as 25 TU in different regions of the world and exhibiting highly distinctive geospatial patterns. The known latitudinal gradient in ^3^H for precipitation extending from the tropics to the poles was accurately predicted, as well as substantially higher ^3^H activities around the poles. The northern hemisphere showed significantly higher predicted ^3^H activities than the lower latitudes, equatorial areas, and the southern hemispheric land masses.

**Figure 2:**
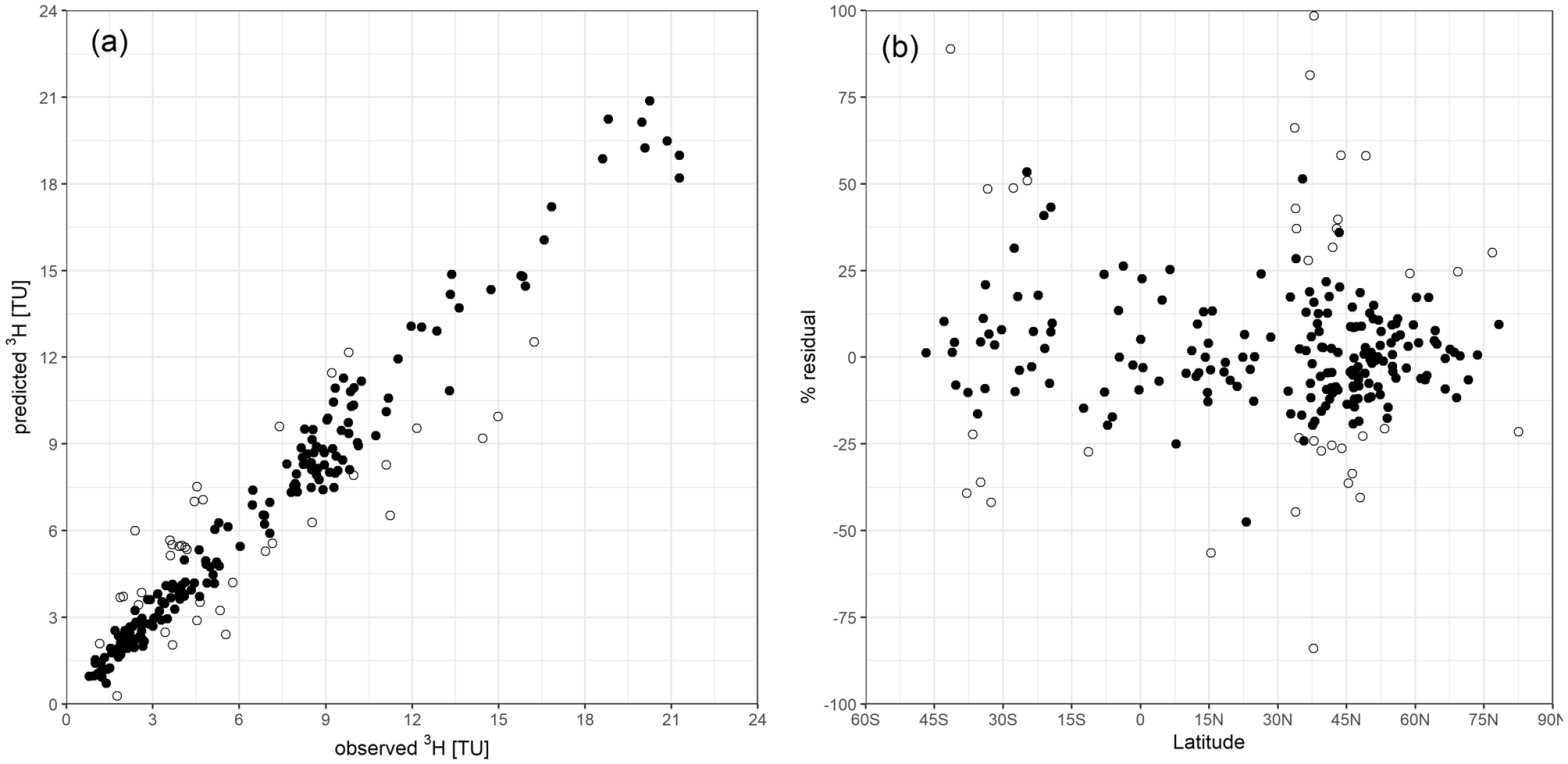
^3^H predictive model validation. Left: predicted vs observed contemporary ^3^H contents in global precipitation expressed in tritium units (TU). Right: model ^3^H residuals expressed as percent deviation between predicted and observed mean tritium values vs latitude. Open circles indicate data points with biases > 20% or beyond the MAB. Figure created in R 4.1.0 (with ggplot2 3.3.5 library, all https://cran.r-project.org/).

The predicted distinctive spatial global patterns depart from a commonly held belief that ^3^H in precipitation in the post-bomb timeframe would be uniformly low and near or below analytical detection limits worldwide. To affirm our predicted spatial patterns, we performed validation of the prediction model against ^3^H records from selected stations as depicted in Fig. 2. For example, the predicted ^3^H content for 233 stations plotted versus their measured ^3^H data showed our predictions were accurate and in a narrow band within ± 20% of station-based observational data, and thereby validating our model prediction results. The outliers in Fig. 2 are from continental mid-latitude areas and mainly stem from minor local anthropogenic emissions that could not be captured in the initial data screening. The mean absolute bias (MAB) of the model point-based prediction was 0.8 TU, and the absolute percentage bias was 17%. However, we did not find any systematic relationship of bias to latitude or the observed ^3^H values.

Due to the relatively small dataset for modelling and the associated technical challenges to spatially split into calibration and validation subsets, we used several techniques to cross-validate our results. First, we tested our post-bomb ^3^H predictions against pre-bomb proxy ^3^H archives (wines and an ice core) and post-2018 precipitation ^3^H data with monthly records spanning < 3 years, which precluded them from the predictive model due to incorporation criteria; we found predictions matched both the archive and post-2018 data within reasonable uncertainty margins (see discussion in Supplementary Material SM3). Second, we assessed the plausibility of the predicted ^3^H content and isotopic gradients of the continentality effects by comparing our predictions along continentality gradients to measured “continentality endmember” stations (e.g., Yakutsk, Russian Federation), which show the highest ^3^H levels in all available time series from the bomb peak to present, and by considering the ratios of oceanic vs. continental ^3^H levels as a constant during the bomb peak and tail periods. This model confirmation analysis is presented in detail in Supplementary Material SM4.

## Unique global spatial ^3^H patterns

Contemporary Eurasian precipitation follows the well-known ^3^H enrichment gradient^12,24^ concomitant with continentality and the decreasing degree of dilution with maritime water vapour along a west–east transect, revealing the importance of the westerlies which explain the predicted lower east–west gradient from the Pacific to Central Asia^24^. The ^3^H ratios of continental GNIP stations compared to the oceanic end member of long-term GNIP station Valentia (Ireland) on the Atlantic coast for historic, present, and predicted data showed the continental ^3^H end member for Central Eurasia is ca. 22 TU. Our model predictions exceeded 22 TU in some mountainous areas; yet these upper predictions need to be verified with observational data. Our model predicted elevated ^3^H, exceeding 15 TU, over the Himalayas and the Tibetan Plateau in agreement with excesses in cosmogenic radionuclides such as ^35^S^25^ but predicted values also need to be validated with ^3^H data for extreme altitudes. Similarly, the North American continent model exhibited patterns of higher ^3^H levels following a continentality gradient that closely mirrors the spatial distribution of stable water isotopes (*δ*^2^H, *δ*^18^O), clearly demarcating the polar vs. Caribbean influences^26^, and a south-north gradient with markedly lower ^3^H values along the fringes of the Atlantic and Pacific coastlines. These distinctive ^3^H patterns reflect the diluting influence of the presumably low-^3^H Gulf Stream and North Pacific currents, respectively. In the absence of sufficient observational data and stations in general, the location and magnitude of the North American continentality end member remains to be validated. Our model predictions, however, fully agreed with observed ^3^H data from the Sonoran and Chihuahua deserts^27^. Along similar latitudes to North America, the Mediterranean region was distinctive by an absence of open-ocean moisture ^3^H dilution, yet still affected by the rising limb of cosmogenic tritium production spanning from annual mean ^3^H levels around 2.5 TU in Gibraltar up to 6 TU in the Middle East region, along both latitudinal and longitudinal gradients. Our predictions affirmed that higher than expected ^3^H values in subtropical areas are related to deep convective precipitation processes, such as in the South America Chaco-Pampa region (peaking at 8 TU) and in parts of the Sahara. In these regions the coexistence of sufficiently deep high-altitude convection and elevated cosmogenic tritium at higher atmospheric levels is the most likely explanation for these patterns ^28^. In contrast, much of South America, Africa, the Indian subcontinent, Australia, and the Asia–Pacific regions were predicted (and verified) to have low ^3^H levels between ca. 2–4 TU. For some regions, like Central America, the Amazon basin, and the large archipelagos in Southeast Asia and the western Pacific, our model predicted ^3^H contents < 1 TU in precipitation which is at or below the analytical detection limits of most tritium laboratories^29^, thereby posing significant challenges for practical applications of ^3^H as a natural radiotracer for hydrology in these areas. Marine coastal fringes for all continents exhibited lower ^3^H in precipitation compared to inland and is clearly influenced by low-^3^H oceanic moisture impacts; yet the continental gradients are not impacted as much as higher latitudes.

In summary, our new predictive model for ^3^H in modern global precipitation revealed highly distinctive spatial patterns that for many parts of the world, and contrary to widely held expectations, showed significantly higher tritium activities than were anticipated. These findings support the promotion and further development of tritium as a powerful short-lived natural radiotracer in hydrological and environmental science applications, particularly for mapping recently recharged groundwater, critical zone hydrology, and groundwater vulnerability. With improved ^3^H detection technologies (~ 0.1 TU precision) by ^3^He-ingrowth or higher degrees of sample pre-enrichment, tritium coupled to other tracers will support the improvement of spatiotemporal hydroclimatic models. Moreover, our predictive maps provide reliable ^3^H input functions for hydrologic transport models and provide an accurate predictive baseline upon which to detect and assess potential impacts anthropogenic nuclear activities. Model prediction accuracy and precision will be improved in time with further attention to systematic ^3^H in precipitation sampling in data deficient regions.

## Methods

### ^3^H data in precipitation

The geographical distribution of ^3^H sampling sites is shown in Fig. 1. Monthly ^3^H precipitation composite data was obtained from the GNIP database and related publications (IAEA/WMO, 2020) covering the period 2008–2018 (see additional details in Supplementary Materials SM1). This timeframe was selected to represent present-day tritium levels (i.e. post-bomb)^11^ covering an 11-year period falling mostly into solar cycle 24 for at least a part of the station data used^18^. The dataset also included ^3^H data from 5400 monthly precipitation samples at 91 sites not yet published in the GNIP database. Additional data was taken from the literature. The tritium data for our spatial analysis and modeling contained records from 233 sites from 2008 to 2018, but some stations have variable lengths of records (Supplementary material SM1). During our data curation we considered each station regarding its time-dependent position in the solar cycle. Recent studies suggest that the solar cycles 10–12 years impart ca. ± 20% of fluctuation in Central Europe that gets overprinted on annual ^3^H seasonality^17,18^. In our analysis we found that ^3^H records with at least three years of continuous monthly sampling, whether during the rising or falling limb of the solar cycle, corresponded within the ^3^H measurement uncertainty to the stations’ mean value for the period 2008–2018. For our mapping purposes we applied a lower ^3^H detection limit cutoff as < 0.5 TU which is in accordance with the lower limit of international laboratory performance^29^ but also corresponds to the lowest values of recent GNIP samples analyzed (from Galapagos and Ascension Islands).

### Regressor datasets

Our three prediction models used the regressors used in the Regionalized Cluster-Based Water Isotope Prediction 2 (RCWIP2^21,22^) model for *δ*^18^O and *δ*^2^H along with geographic and climatic regressors. The geographic regressors were (weighted) latitude, longitude, elevation, distance to coast and land mass fraction. Climatic regressors included air temperature, vapour pressure, precipitation amount, precipitable water, convective precipitation, wind speed and the continentality index^30^. Where in-situ measurements for the explanatory variables were unavailable they were obtained from the Global Historical Climate Network (GHCN)^31^ or NOAA-NCEP reanalysis data ((https://www.ncep.noaa.gov/). As gridded data for these regressors we used CRU-CL 2.1^32^ and up-sampled NCEP datasets.

### Climatic zone clustering and statistical processing

The ^3^H mapping used the code of RCWIP2, including the fuzzy clustering schema in 36 extra-Antarctic clusters of spatial proximity and climatic similarity^22,23^. As only a fraction of the full clustering scheme (16 of 36 clusters) could be populated with sufficient ^3^H data to derive a full regional model, we allowed for fallback on the tropical or extratropical regression models (Eqs. 2, 3). A spatial coverage map of regionalized tropical and extratropical models is given in Supplementary Material SM2. The RCWIP2 algorithms automatically detected the best-fitting regression equation and covariates^23^. However exceptionally, the use of the tropical and extratropical models had to be enforced for a few clusters to avoid runaway outlier ^3^H predictions, which we attributed to the spatially and climatically skewed distribution of ^3^H input data (Supplementary Table S2).

## Supplementary Information

Supplementary Information.

## Data Availability

Numerical ^3^H data from GNIP are available online at: https://nucleus.iaea.org/wiser. The ^3^H dataset used for regression analysis, and the resulting maps and grids are available at: https://isotopehydrologynetwork.iaea.org. Other sources of ^3^H data are listed in the supplementary materials.

## References

[1] Gat, J. R. The isotopes of hydrogen and oxygen in precipitation. in *Handbook of Environmental Isotope Geochemistry* (eds. Fritz, P. & Fontes, J.Ch.). Vol. 1. 21–47. (Elsevier, 1980).

[2] Ferronsky V, Polyakov V (1982). Origin of hydrosphere of Earth in the light of isotopic and theoretical studies. Geokhimiya.

[3] Clark ID, Fritz P (1997). Environmental Isotopes in Hydrogeology.

[4] Jasechko S (2017). Global aquifers dominated by fossil groundwaters but wells vulnerable to modern contamination. Nat. Geosci..

[5] Gleeson T (2016). The global volume and distribution of modern groundwater. Nat. Geosci..

[6] Maloszewski, P. & Zuber, A. Lumped parameter models for the interpretation of environmental tracer data. in *Manual on Mathematical Models in Isotope Hydrogeology*. 9–58. (International Atomic Energy Agency, 1996).

[7] Gröning M, Rozanski K (2003). Uncertainty assessment of environmental tritium measurements in water. Accred. Qual. Assur..

[8] Masarik J, Beer J (2009). An updated simulation of particle fluxes and cosmogenic nuclide production in the Earth's atmosphere. J. Geophys. Res. Atmos..

[9] Rozanski K, Gonfiantini R, Araguas-Araguas L (1991). Tritium in the global atmosphere: distribution patterns and recent trends. J. Phys. G Nucl. Part. Phys..

[10] *Canadian Nuclear Safety Commission, Standards and Guidelines for Tritium in Drinking Water (INFO-0766)* (2014).

[11] Morgenstern U, Taylor CB (2009). Ultra low-level tritium measurement using electrolytic enrichment and LSC. Isot. Environ. Health Stud..

[12] Roether, W. Estimating the tritium input to groundwater from wine samples: Groundwater and direct run-off contribution to Central European surface waters. in *Isotopes in Hydrology*. 73–91. (International Atomic Energy Agency, 1968)

[13] Schlosser P (1988). Tritium/^3^He dating of shallow groundwater. Earth Planet. Sci. Lett..

[14] van Rooyen JD, Watson AP, Miller JA (2020). Combining quantity and quality controls to determine groundwater vulnerability to depletion and deterioration throughout South Africa. Environ. Earth Sci..

[15] Li Z, Si B (2018). Reconstructed precipitation tritium leads to overestimated groundwater recharge. J. Geophys. Res. Atmos..

[16] West JB (2009). Isoscapes: Understanding Movement, Pattern, and Process on Earth Through Isotope Mapping.

[17] Kern Z (2020). Isoscape of amount-weighted annual mean precipitation tritium (3H) activity from 1976 to 2017 for the Adriatic-Pannonian region—AP3H_v1 database. Earth Syst. Sci. Data.

[18] László E, Palcsu L, Leelőssy Á (2020). Estimation of the solar-induced natural variability of the tritium concentration of precipitation in the Northern and Southern Hemisphere. Atmos. Environ..

[19] Borković D, Bronić IK (2021). Solar activity cycles recorded in long-term data on tritium activity concentration in precipitation at Zagreb. Croatia. Radiat. Phys. Chem..

[20] International Atomic Energy Agency. *Global Network of Isotopes in Precipitation. The GNIP Database. Vienna: International Atomic Energy Agency*. https://nucleus.iaea.org/wiser . Accessed 10 Oct 2019.

[21] Fourré E (2006). Past and recent tritium levels in Arctic and Antarctic polar caps. Earth Planet. Sci. Lett..

[22] Terzer S (2013). Global isoscapes for *δ*^18^O and *δ*^2^H in precipitation: Improved prediction using regionalized climatic regression models. Hydrol. Earth Syst. Sci..

[23] Terzer-Wassmuth S (2021). Improved high-resolution global and regionalized isoscapes of δ18O, δ2H, and d-excess in precipitation. Hydrol. Process..

[24] Ferronsky VI, Polyakov VA (1982). Environmental isotopes in the hydrosphere.

[25] Lin M (2016). Resolving the impact of stratosphere-to-troposphere transport on the sulfur cycle and surface ozone over the Tibetan Plateau using a cosmogenic ^35^S tracer. J. Geophys. Res. Atmos..

[26] Thatcher LL (1962). The distribution of tritium fallout in precipitation over North America. Hydrol. Sci. J..

[27] Eastoe CJ (2012). Future use of tritium in mapping pre-bomb groundwater volumes. Groundwater.

[28] Tremoy G (2014). Clustering mesoscale convective systems with laser-based water vapor *δ*^18^O monitoring in Niamey (Niger). J. Geophys. Res. Atmos..

[29] Copia L (2020). Proficiency testing of 78 international laboratories measuring tritium in environmental waters by decay counting and mass spectrometry for age dating and water resources assessment. Rapid Commun. Mass Spectrom..

[30] Conrad V (1946). Usual formulas of continentality and their limits of validity. EOS Trans. Am. Geophys. Union.

[31] Peterson TC (1998). Global historical climatology network (GHCN) quality control of monthly temperature data. Int. J. Climatol..

[32] New M, Lister D, Hulme M, Makin I (2002). A high-resolution data set of surface climate over global land areas. Clim. Res..

