# Supplementary material for: High spatial resolution prediction of tritium (3H) in contemporary global precipitation
Source: Sci Rep. 2022 Jun 17;12:10271. doi: 10.1038/s41598-022-14227-5 (PMC9205854; doi:10.1038/s41598-022-14227-5)
Supplement: Supplementary file 1 — Supplementary Information. [file 41598_2022_14227_MOESM1_ESM.pdf]

High Spatial Resolution Prediction of Tritium (^3H) in Contemporary Global Precipitation

Supplementary Materials

S. Terzer-Wassmuth, L. Araguás-Araguás, L. Copia and L.I. Wassenaar

SM1 Data selection and multi-year scaling

We based our ^3H data selection for mapping purposes on the last 11-year neutron flux (NF) cycle (2008-18) following [1]. Neither cut-off date is located exactly in the peak or the valley of the solar cycle 24, the last cosmogenic neutron cycle. Both cut-off years exhibit a neutron flux excess of 4.5-5% compared to the long-term mean (1965-2019) value. The choice of ^3H data was further governed by practical considerations of data availability, i.e., the laboratory processing and turnover times, and because samples for 2019 and beyond were not available due to the COVID-19 pandemic.

According to [1] and [2], the temporal evolution of the global ^3H distribution entered the post-bomb phase after ca. 1990 in the southern hemisphere, and ca. in 2000 in the northern hemisphere. Therefore, we deemed it reasonable to define the weighted mean value of the “first post-peak solar cycle”, i.e., covering solar cycle 24 with the mentioned shift of one year. Notably, the NF of solar cycle 24 was above the 1965-2019 average (i.e., solar cycles 20-24), with only the lowest fluxes in 2014 and 2015 being slightly below the grand long-term mean of the multi-decade observation period.

Nonetheless, we estimated a ^3H “multi-year scaling factor” for every year between 2008 and 2018 relative to the mean of the observation period based on n=15 stations whose record covered the full 2008-18 period (Table 1). Though there seemed to be a difference between N-S hemispheres, we opted for global scaling factors due to sparse and unevenly distributed data from the southern hemisphere. As data collection and interpretation beyond 2018 is only done for the Vienna station, Fig. S1 shows the extension of the scaling factor series for this station; however, given the large fluctuations around the mean of stations in the period before that these results should be interpreted with caution.

**Annual mean TU compared to mean 2008-18**													
**Station**	**Latitude**	**2008**	**2009**	**2010**	**2011**	**2012**	**2013**	**2014**	**2015**	**2016**	**2017**	**2018**	**2019**	**2020**
Ny Alesund	78.25 N	1.21		1.25	1.32	0.84		1.04	0.87	0.83	0.99	0.91		
Reykjavik	64.13 N	1.00	1.21	1.05	1.05	0.88	1.04	1.06	0.90	0.87	0.98	1.07		
Kuopio	62.89 N	1.11	1.15	1.03	1.00	1.07	0.97	1.00	0.92	0.97	0.87	1.00		
Valentia	51.93 N	1.15	1.12	1.15	0.92	1.14	0.96	0.89	0.88	0.99	0.91	0.90		
Vienna	48.25 N	1.12	1.16	1.18	1.00	0.88	0.94	0.96	0.75	0.85	0.89	1.06	1.01	0.91
Ponta Delgada	37.77 N	1.15	0.98	1.11	1.01	1.04	0.95	1.00	0.97	0.87	0.95	0.90		
Havana*	23.05 N	0.95	1.25	1.06	1.11	1.17	0.97	0.84	0.90	1.01	0.83			
Hong Kong	22.32 N	1.08	1.28	1.08	1.00	1.13	0.93	0.99	0.89	0.88	0.83	1.05		
Diliman Q. City*	14.64 N	0.95	1.17	1.23	0.94	1.08	1.01	1.02	0.88	0.97	0.73			
Bangkok	13.73 N	1.19	1.33	0.87	1.07	0.98	0.91	0.85	0.75	0.82	0.91	1.13		
Belo Horizonte*	19.87 S		1.22	1.04	0.92	0.99	0.99	0.79	0.97	1.00	0.90	1.07		
Isla de Pascua	27.17 S	1.26	1.03	1.22	1.02	0.89	1.06	0.94	0.79	1.05	0.89	0.81		
Kaitoke*	41.10 S	1.06	1.15	1.08	1.04	1.00	0.92	0.88	0.89	1.01	0.96			
Puerto Montt	41.47 S	1.18	1.05	1.08	1.08	0.98	0.77	0.90	0.90	1.13	0.94	1.05		
Halley Bay*	75.58 S	1.06		0.92	0.96	1.02	0.98	1.05	1.01	1.02				
	rTU	1.10	1.16	1.09	1.03	1.01	0.96	0.95	0.88	0.95	0.90	1.00		

Global Average scaling factor	SD	0.09	0.10	0.11	0.10	0.10	0.07	0.08	0.07	0.09	0.07	0.10		
Northern Hemisphere scaling factor	rTU	1.09	1.18	1.10	1.04	1.02	0.96	0.97	0.87	0.91	0.89	1.00		
	SD	0.09	0.10	0.11	0.11	0.12	0.04	0.08	0.07	0.07	0.08	0.09		
Southern Hemisphere scaling factor	rTU	1.14	1.11	1.07	1.00	0.98	0.94	0.91	0.91	1.04	0.92	0.98		
	SD	0.10	0.09	0.10	0.06	0.05	0.11	0.09	0.08	0.05	0.03	0.15		
Oulu NF		1.03	1.05	1.02	0.99	0.97	0.96	0.95	0.95	1.00	1.02	1.03	1.04	1.05

Table S1 – Annual ^3H levels relative to the 2008-18 mean for selected stations. Stations marked with an asterisk suffered from gaps in the time series or data not yet available at the time of writing.

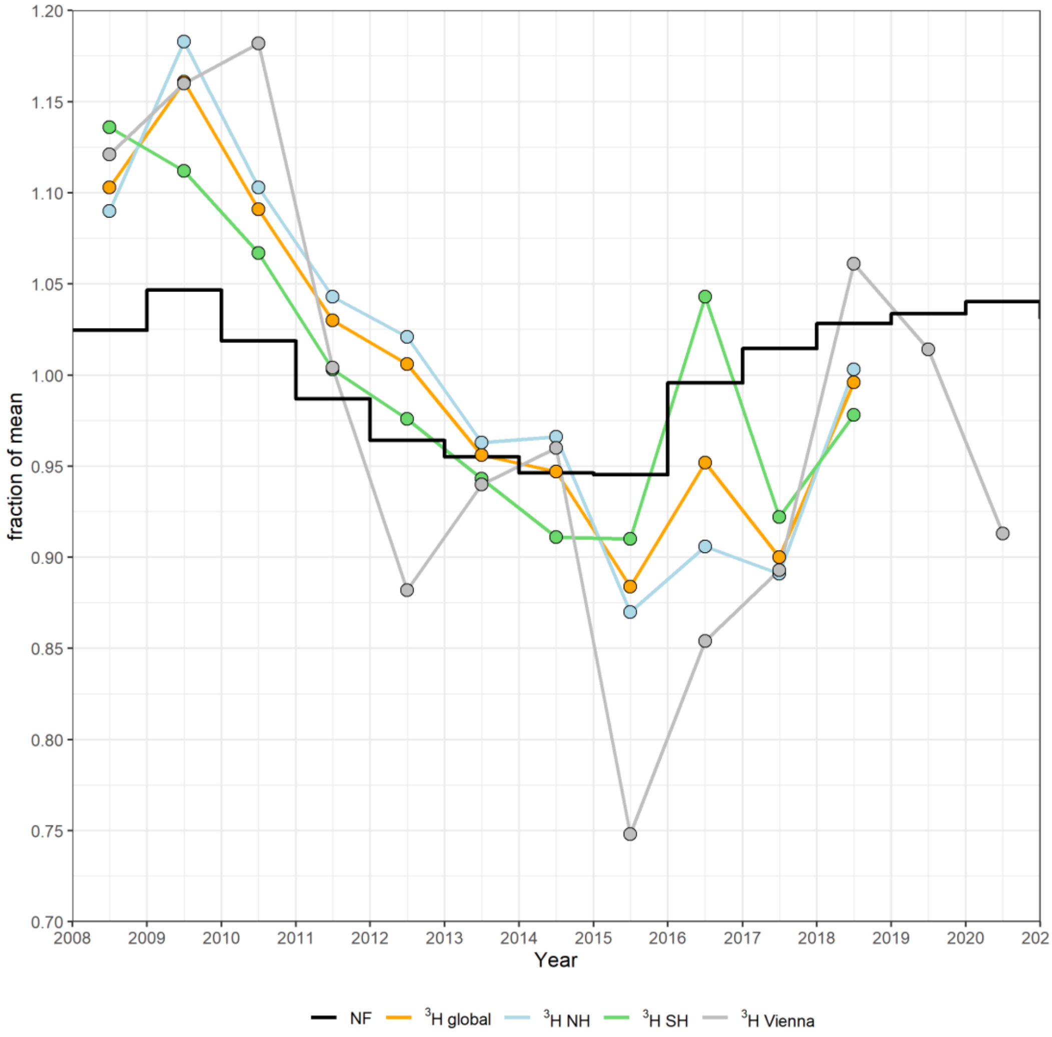

Figure S1: Time series of the cosmogenic neutron flux at Oulu observatory 2008-2020 (https://cosmicrays.oulu.fi/), in corrected counts × min^{-1}) and ^3H levels for global, northern hemispheric and southern hemispheric distributions and Vienna reference station (in TU). All data are normalized to the mean values of 2008-18. ^3H levels are plotted as dots mid-year for better legibility. Figure created in R 4.1.0 (with ggplot2 3.3.5 library, all https://cran.r-project.org/).

Given the comparably high NF during the valley years 2014-15, it remains unclear whether these years represent an “absolute minimum” of ^3H input to be expected under natural steady-state conditions. It is obvious that this question can only be answered by longer multi-decadal sampling under current conditions of low anthropogenic ^3H input to the global hydrological cycle, anticipating a stronger sunspot maximum (and hence lower NF) in the middle of the cycle.

Data selection was performed so that preferably multi-year records covering a minimum number of observations within the 2008-18 observation period were included. However, even in the case where shorter records were included, their offset compared to the 2008-18 grand mean should not exceed ±10% (there are no short-term records from 2009/10 alone, which would violate this criterion). We deemed this a reasonable trade-off between data scarcity and model selection tightness, especially since most laboratory analytical methods give a relative ^{3}H error of ±10%.

Additional supplementary tables:

- List of observed data points and geographical/climatological covariates – see references [3-19]

SM2 Additional information on regression modelling

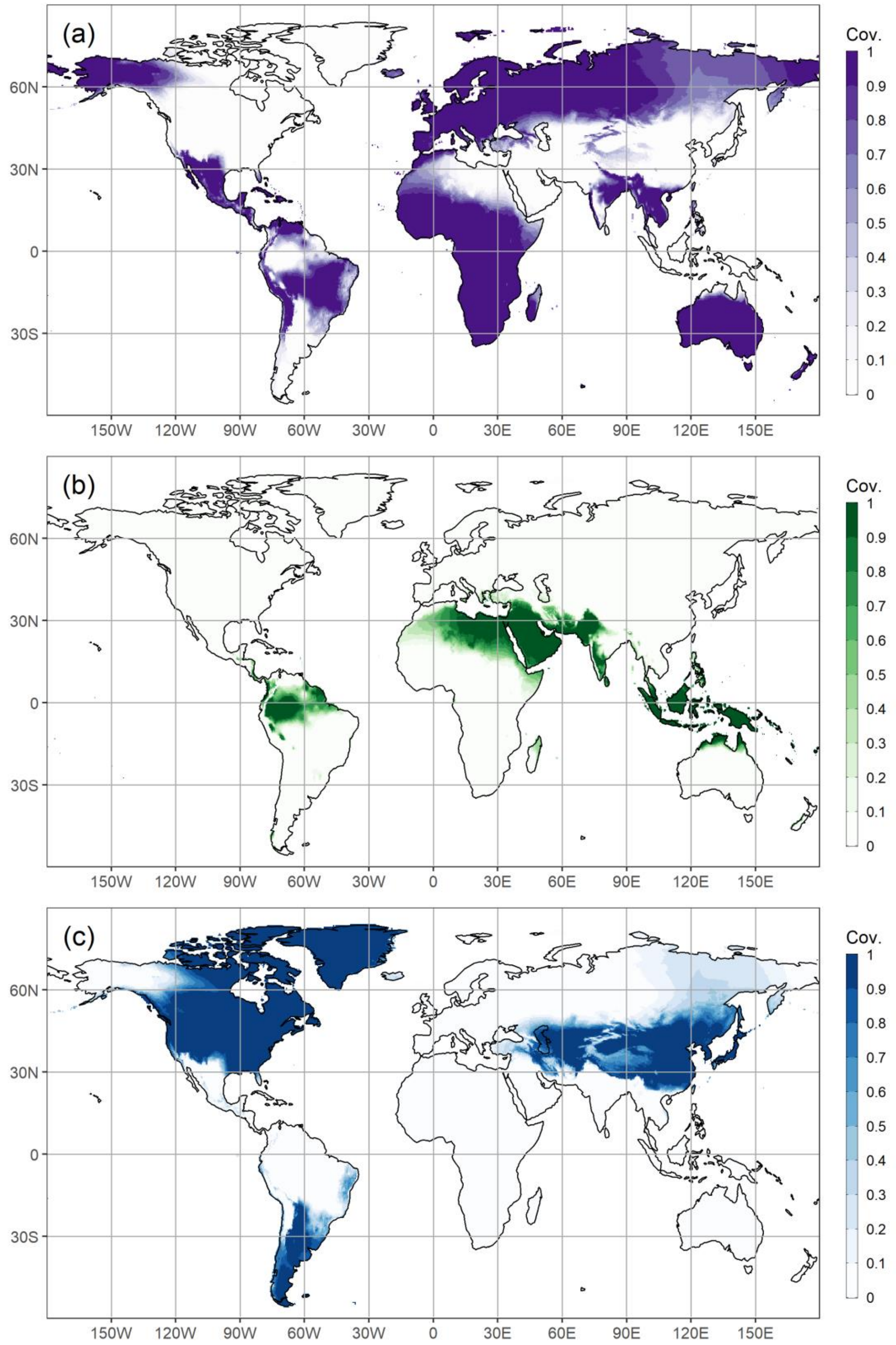

Figure S2: Coverage fractions of (a) cluster-specific, (b) tropical and (c) extratropical replacement models used for regression modelling. Figure created in R 4.1.0 (with ggplot2 3.3.5, raster 3.4-13 and rgdal 1.5-23 libraries, all https://cran.r-project.org/).

Cluster[1)]	Type[2)]	Model string	R^2	RMSE	Coefficients														
					Intercept	PP	AT	VP	PW	LONG	LONG2[3)]	ALT	CPN	LHF	NLR	OLR	WS	wLT	DTC	LMF
**0**	**G**	**H3~wLT+DTC+AT+LMF+LONG+NLR**	**0.79**	**2.23**	**61.396**		**-0.208**			**0.006**					**-0.028**			**0.031**	**2.56×10^{-6}**	**6.207**
**39**	**E**	**H3~AT+LMF+DTC+ALT+wLT+LONG**	**0.81**	**2.19**	**71.711**		**-0.250**			**0.005**		**-0.001**						**0.018**	**3.04×10^{-6}**	**8.015**
**40**	**T**	**H3~wLT+LMF+AT+OLR+CPN+PP**	**0.62**	**1.20**	**9.439**	**0.000**	**0.006**						**-0.006**			**-0.040**		**0.051**		**2.282**
1	C	H3~LMF+LONG+OLR+CPN	0.82	1.11	20.940					0.081			-0.030			-0.080				7.679
2	C	H3~LONG+DTC+NLR+LMF+PW+wLT	0.91	1.12	10.174				-0.407	0.047					-0.055			0.014	2.42×10^{-6}	7.610
3	C	H3~LMF+VP+wLT+LONG	0.97	0.81	12.984			-1.852		-0.016								-0.053		25.731
4	E	H3~AT+LMF+DTC+ALT+wLT+LONG	0.81	3.68	71.711		-0.250			0.005		-0.001						0.018	3.04×10^{-6}	8.015
5	T	H3~wLT+LMF+AT+OLR+CPN+PP	0.62	0.73	9.439	0.000	0.006						-0.006			-0.040		0.051		2.282
6	C	H3~LHF+LONG2+LMF	0.98	0.14	5.453						-0.018			-0.045						0.385
7	E	H3~AT+LMF+DTC+ALT+wLT+LONG	0.81	3.50	71.711		-0.250			0.005		-0.001						0.018	3.04×10^{-6}	8.015
8	E	H3~AT+LMF+DTC+ALT+wLT+LONG	0.81	1.54	71.711		-0.250			0.005		-0.001						0.018	3.04×10^{-6}	8.015
9	E	H3~AT+LMF+DTC+ALT+wLT+LONG	0.81	1.64	71.711		-0.250			0.005		-0.001						0.018	3.04×10^{-6}	8.015
10	C	H3~LONG+OLR+CPN	0.71	0.85	26.485					0.113			-0.052			-0.086				
11	C	H3~LHF+DTC+PW	0.97	0.22	5.610				-0.052					-0.020					1.34×10^{-6}	
12	T	H3~wLT+LMF+AT+OLR+CPN+PP	0.62	1.86	9.439	0.000	0.006						-0.006			-0.040		0.051		2.282
13	C	H3~DTC+AT	0.95	0.15	-19.337		0.068												1.17×10^{-6}	
14	C	H3~NLR+LHF	0.92	0.26	-6.644									0.036	0.101					
15	C	H3~LHF+ALT	0.81	0.19	2.372							0.000		-0.009						
16	C	H3~AT+DTC+wLT+ALT	0.98	0.62	135.800		-0.451					-0.003						-0.036	1.17×10^{-5}	
17	E	H3~AT+LMF+DTC+ALT+wLT+LONG	0.81	2.53	71.711		-0.250			0.005		-0.001						0.018	3.04×10^{-6}	8.015
18	E	H3~AT+LMF+DTC+ALT+wLT+LONG	0.81	2.06	71.711		-0.250			0.005		-0.001						0.018	3.04×10^{-6}	8.015
19	E	H3~AT+LMF+DTC+ALT+wLT+LONG	0.81	NaN	71.711		-0.250			0.005		-0.001						0.018	3.04×10^{-6}	8.015
20	E	H3~AT+LMF+DTC+ALT+wLT+LONG	0.81	2.20	71.711		-0.250			0.005		-0.001						0.018	3.04×10^{-6}	8.015
21	E	H3~AT+LMF+DTC+ALT+wLT+LONG	0.81	1.83	71.711		-0.250			0.005		-0.001						0.018	3.04×10^{-6}	8.015
22	E	H3~AT+LMF+DTC+ALT+wLT+LONG	0.81	1.46	71.711		-0.250			0.005		-0.001						0.018	3.04×10^{-6}	8.015
23	E	H3~AT+LMF+DTC+ALT+wLT+LONG	0.81	2.30	71.711		-0.250			0.005		-0.001						0.018	3.04×10^{-6}	8.015
24	C	H3~NLR+OLR	0.67	1.06	9.283										0.081	-0.050				
25	C	H3~LMF+PW+ALT	0.91	0.15	1.981				-0.028			0.000								0.973
26	C	H3~NLR+ALT+OLR	0.98	0.14	4.754							0.000			0.088	-0.033				
27	T	H3~wLT+LMF+AT+OLR+CPN+PP	0.62	0.52	9.439	0.000	0.006						-0.006			-0.040		0.051		2.282
28	C	H3~LONG+wLT	0.72	0.72	8.191					0.081								0.067		
29	E	H3~AT+LMF+DTC+ALT+wLT+LONG	0.81	1.85	71.711		-0.250			0.005		-0.001						0.018	3.04×10^{-6}	8.015
30	E	H3~AT+LMF+DTC+ALT+wLT+LONG	0.81	2.27	71.711		-0.250			0.005		-0.001						0.018	3.04×10^{-6}	8.015
31	T	H3~wLT+LMF+AT+OLR+CPN+PP	0.62	0.28	9.439	0.000	0.006						-0.006			-0.040		0.051		2.282
32	T	H3~wLT+LMF+AT+OLR+CPN+PP	0.62	0.72	9.439	0.000	0.006						-0.006			-0.040		0.051		2.282
33	T	H3~wLT+LMF+AT+OLR+CPN+PP	0.62	0.22	9.439	0.000	0.006						-0.006			-0.040		0.051		2.282
34	C	H3~VP+wLT+WS	0.98	0.08	5.134			-0.139									-0.103	-0.048		
35	C	H3~VP+PP+LONG2+WS+DTC	0.95	0.13	6.280	-0.001		-0.173			0.017						-0.118		-8.86×10^{-7}	
36	T	H3~wLT+LMF+AT+OLR+CPN+PP	0.62	0.52	9.439	0.000	0.006						-0.006			-0.040		0.051	2.56×10^{-6}	2.282

Table S2: Regression equations used for the climatic clusters. [1)] clusters 0, 39 and 40 denote global and zonal extratropical/tropical models. [2)] G = global, T = tropical, E = extratropical, C = cluster-specific. [3)] the LONG2 variable is an inverted longitude value: LONG2=IFELSE(LONG>0, LONG-180, LONG+180). This was used for clusters whose centroid is located between 90°E and 90°W to avoid misleading regression choice due to the date line.

SM3 Historical ^3H proxies versus recent data

Under the assumption that contemporary steady-state ^3H levels are similar to the pre-bomb values, we compared our predicted ^3H values with proxy ^3H information dated from between 1935 and 1954. Most of these proxies were vintage wines, and one ice core from the Canadian Arctic [20]. For the reconstruction of ^3H levels from vintage wines, both the geographical location of the samples within the respective continent, as well as the plant physiology needs to be considered. Firstly, many of the wines commonly used as references stem from locations under important maritime influence and hence, lower ^3H levels may be expected than those sampled in more continental sites. Secondly, the tritium uptake into grapes is seasonally biased towards the weeks before harvest; hence late harvests may sample ^3H from below the annual mean in precipitation. Thirdly, one must be aware of the technological differences between the measurements done at the early days of tritium analysis compared to nowadays. Table S3.1 shows the proxies used.

**Vintage Wines**						
**Location**	**Source**	**Period**	**^3H**	**σ^3H**	**Pred. ^3H**	**Remark**
Germany	[21]	1949-52 avg	5.9	0.6	7.3	*
Valentia estimate	[21]		3.0	1.5	2.9	
Gironde	[22]	1928-45 avg	4.4	0.7	4.8	
Rhone	[22]	1929-51 avg	3.8	0.7	6.0	*
Jerez	[23]	1942-51 avg	3.1	0.4	3.0	
New York	[22]	1940-52 avg	5.9	0.4	5.5	
South Africa	[24]	1949-54 avg	4.2		2.6	**
		measured?	1.2		2.6	**
Australia	[25]	1942-53 avg	2.7	1.1	2.7	
**Ice core data**						
Agassiz ice cap	[20]	1935-44	12.5	1.9	9.4	

Table S3.1: Overview of pre-bomb proxies used for comparison with the ^3H isoscape (- late harvest; ^3H levels may have been below annual mean value; ** - two values given; unclear which value was proposed for usage by authors)*

Notwithstanding the above constraints, Figure S3 shows reasonable agreement for the majority of the historical ^3H data points. To further test the predictive performance of our model, we compiled mean ^3H contents (unweighted) of some GNIP samples at the IAEA Isotope Hydrology Laboratory (Table S3.2). The overall fit was good for values < 10 TU (though there is limited additional data available for stations > 10 TU). Under-predictions are seen in Southern Africa and continental Argentina. We are aware that multiple constraints may apply for this analysis (unweighted means of observed data, not corrected for their position in the solar cycle, short records yet of different lengths from stations where no previous records exist etc.); however, with the overall improving data availability we are optimistic that a more detailed assessment will be possible soon.

**Station**	**Country**	**Latitude**	**Longitude**	**Year(s)**	**Mean ^3H**	**Pred. ^3H**
Asuncion	Paraguay	-25.3	-57.59	2018-19	4.1	4.8
Cameron Highlands	Malaysia	4.47	101.39	2019	1.3	0.6
Hermosillo	Mexico	29.09	-110.99	2018-19	2.7	4.2
Kharkiv	Ukraine	49.99	36.28	2019-20	9.6	11.3
Kota Kinabalu	Malaysia	5.98	116.15	2019	1.0	1.3
Kuma Konda	Togo	6.96	0.57	2017	2.2	2.1

Los Gigantes	Argentina	-31.39	-64.78	2018-20	9.7	6.4
Luanda	Angola	-8.82	13.23	2020	1.6	1.5
Maroua	Cameroon	10.59	14.32	2020	4.3	4.1
Maseru	Lesotho	-29.31	27.51	2018-20	4.3	2.4
Mongo	Chad	12.19	18.69	2019	5.5	5.1
Niamey	Niger	13.51	2.12	2019-20	4.4	4.7
Oxbow	Lesotho	-28.77	28.63	2018-20	4.4	3.0
Prodromos	Cyprus	34.95	32.84	2018-20	4.6	4.3
Ras Muneef	Jordan	32.38	35.81	2016-19	4.0	4.6
Sarh	Chad	9.15	18.38	2019	4.4	3.4
Winnipeg	Canada	49.89	-97.15	2020	11.1	13.8

Table S3.2: Very recent ^3H measurements (These correspond to new sites in the GNIP measurement programme which have produced data only in 2018 or later). Note - these sites are not scaled because scaling factors are not yet available.

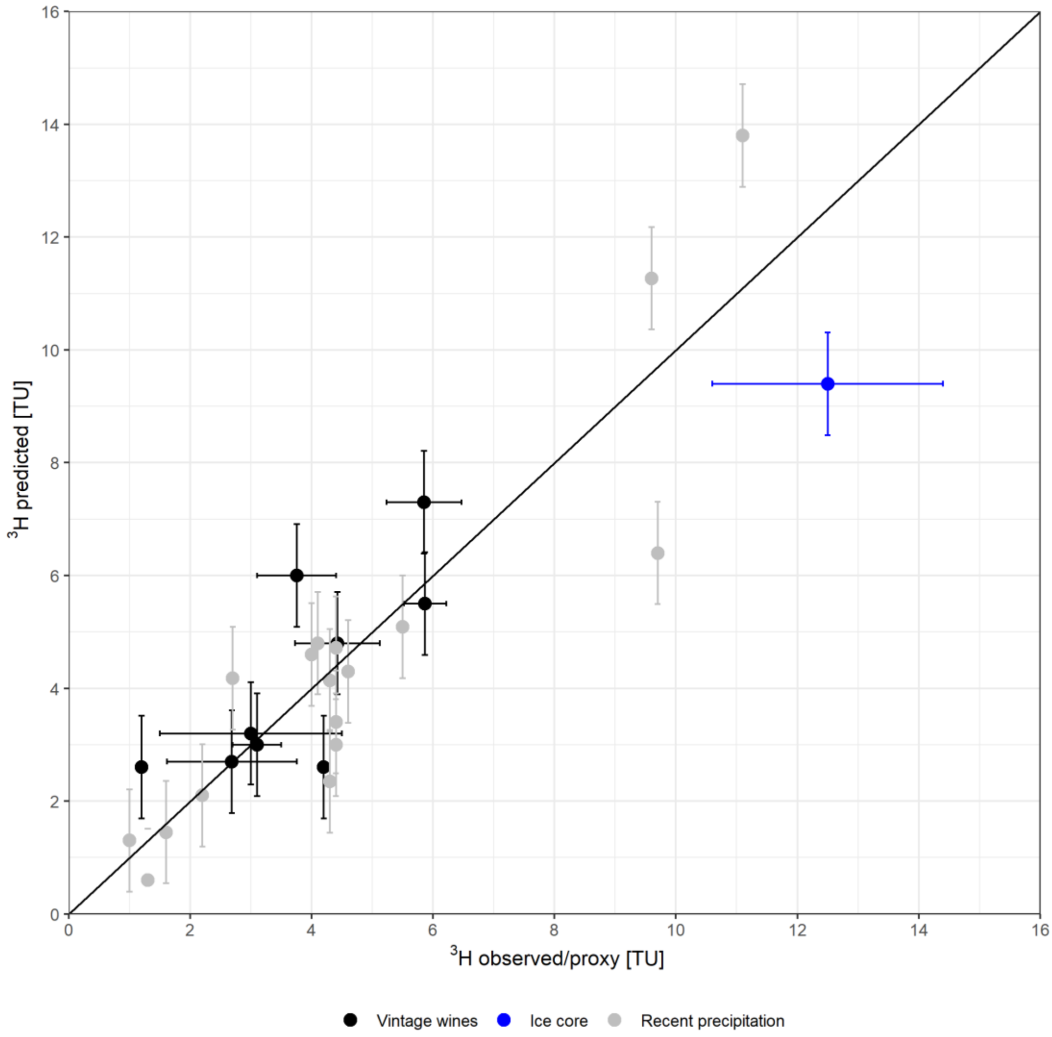

Figure S3: Scatterplot of RCWIP-^3H predictions vs. ^3H proxy data and recently analysed data. Horizontal error bars show analytical uncertainty where reported ("recent precipitation" are mean values of multiple measurements). Vertical error bars indicate the overall isoscape uncertainty of 0.9 TU. Figure created in R 4.1.0 (with ggplot2 3.3.5 library, all https://cran.r-project.org/).

SM4 Continentality analysis

We aimed to delineate the "continentality endmembers" for both the Eurasian and North American land masses through alternative techniques based on recent and past data and related ^{3}H stations along these continentality gradients to them, expressing their ^{3}H levels as fractions of each endmember. We hypothesized that similar fractions for observed and predicted at the same location would reveal a good ability of the model the predict continentality gradients.

Figure S4.1 shows the data points discussed in this section; a transect from Valentia, Atlantic Ocean, to Yakutsk.

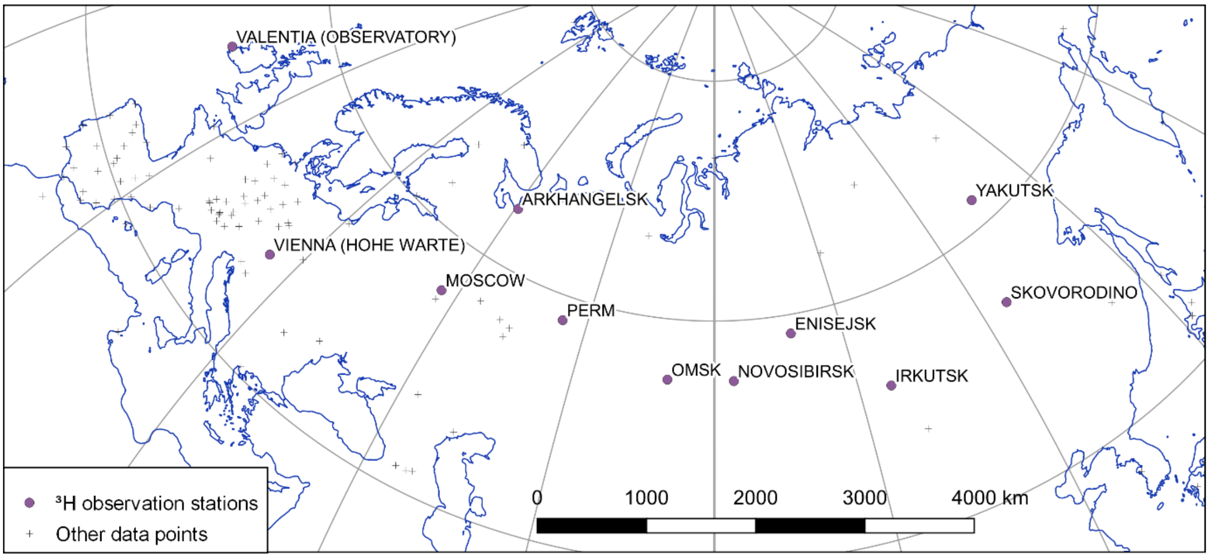

Figure S4.1 Overview map of Eurasia showing the ^{3}H monitoring sites discussed in the text. Figure created in QGIS 3.3 (https://www.qgis.org/).

We compared the annual means during the bomb peak tail (Russia 1969-83, [9]) to our isoscape. Assuming overall stability in macroscale atmospheric processes and therefore homogeneous dispersion of the atmospheric ^{3}H over the continents relative to the global input function, we calculated for each station the multi-year mean fraction of the continentality endmember. We performed a similar analysis for observed data from 2016-18 [14-16] in Russia after solar cycle adjustment, again relating them to the observed ^{3}H levels for Yakutsk. The results are shown in Figure S3.2 which underlines the predictive power of the isoscape for Eurasia. Past, present, and predicted data represent the continentality trend well, though caution has to be exercised at the sites near the Caspian Sea when the distance to coast is derived from geodata treating it as an actual sea, which is an unrealistic assumption in terms of atmospheric circulation. Such blunder results in substantial residuals at GNIP stations like Astrakhan with a distance to the Caspian coast of < 100 km, but 4100 km to the Atlantic moisture source.

Station data			Distance [km]		^{3}H [TU] observed			^{3}H multi-yer scaled			Annual precipitation [mm]			Mean ^{3}H 2016-18		^{3}H relative to Yakutsk		
								0.95[1]	0.90[1]	1.00[1]								
**Name**	**Lat**	**Long**	**Atl.**	**Pacific**	**2016**	**2017**	**2018**	**2016**	**2017**	**2018**	**2016**	**2017**	**2018**	**Obs.** [2]	**Predicted**	**Pres.**	**Past**	**Isoscape**
Valentia	51.90	-10.35	1		2.4	2.2	2.2	2.5	2.5	2.2	1659	1513	1771	2.4	2.9	0.112	0.148	0.148
Vienna	48.21	16.20	1900		8.4	8.8	10.4	8.8	9.7	10.5	521	588	716	9.8	9.9	0.452	0.637	0.507
Kuopio	62.88	27.67	2500		9.5	8.5	9.7	9.9	9.4	9.7	691	727	558	9.7	11.3	0.449		0.579
Kaliningrad	54.70	20.62	2000		11.0	8.9	10.2	11.5	9.9	10.2	803	1078	632	10.5	10.3	0.486		0.528
Moscow Baltschug	55.74	37.61	3100		12.2	12.5	11.7	12.9	13.9	11.7	857	855	649	12.9	13.0	0.600	0.829	0.669
Arkhangelsk	64.58	40.50	3200		12.5	10.6	11.0	13.1	11.8	11.0	545	779	582	11.9	12.0	0.553	0.596	0.613
Gorodets	56.63	43.48	3400		13.8	13.0	11.9	14.5	14.4	11.9	681	632	530	13.7	14.2	0.636		0.727
Tsimlyansk	47.61	42.10	3700		14.5	12.2	12.7	15.2	13.6	12.7	553	456	418	14.0	10.8	0.648		0.556
Cheboksary	56.08	47.33	3700		13.4	13.5	13.7	14.1	15.0	13.8	570	645	520	14.3	14.9	0.664		0.763
Tetyushi	54.95	48.81	3800		15.6	14.0	13.0	16.4	15.5	13.0	485	613	487	15.0	14.3	0.696		0.735
Kazan	55.78	49.18	3800		16.8	14.9	13.0	17.6	16.6	13.0	656	616	483	16.0	14.8	0.741		0.76
Astrakhan	46.27	48.03	4100		15.2	14.9	11.0	15.9	16.6	11.0	373	189	128	15.2	10.0	0.705		0.511
Perm	57.95	56.20	4100		17.0	16.3	14.8	17.9	18.1	14.9	525	805	638	17.0	16.1	0.788	0.737	0.824
Omsk	54.93	73.40	5200		20.6	19.6	22.0	21.6	21.7	22.1	462	345	450	21.8	20.1	1.011	0.916	1.033
Novosibirsk	55.03	82.90	5700		20.7	19.6	20.3	21.7	21.8	20.4	427	502	612	21.2	19.2	0.984	1.021	0.987
Tura	64.17	100.07	5800		18.2	19.6	19.5	19.1	21.7	19.5	289	388	354	20.3	20.2	0.939		1.038
Yeniseysk	58.45	92.15	5900		21.5	19.1	18.6	22.6	21.3	18.7	429	490	419	20.9	20.9	0.968	0.901	1.071
Irkutsk	52.27	104.35	6900		21.0	21.8	20.3	22.0	24.2	20.4	566	472	471	22.2	19.0	1.029	1.236	0.974
Yakutsk	62.08	129.75	6900		20.2	21.7	19.5	21.2	24.1	19.5	228	223	238	21.6	19.5	1.000		1.000
Skovorodino	54.00	124.00	7500	2200	18.9	23.9	20.3	19.8	26.5	20.4	449	562	517	22.5	18.2	1.043	1.065	0.934

Table S3: Observed and predicted ^{3}H data for the Valentia-Yakutsk transect and scaling to continentality endmember Yakutsk. [1] scaling factors applied, [2] rescaled observed data

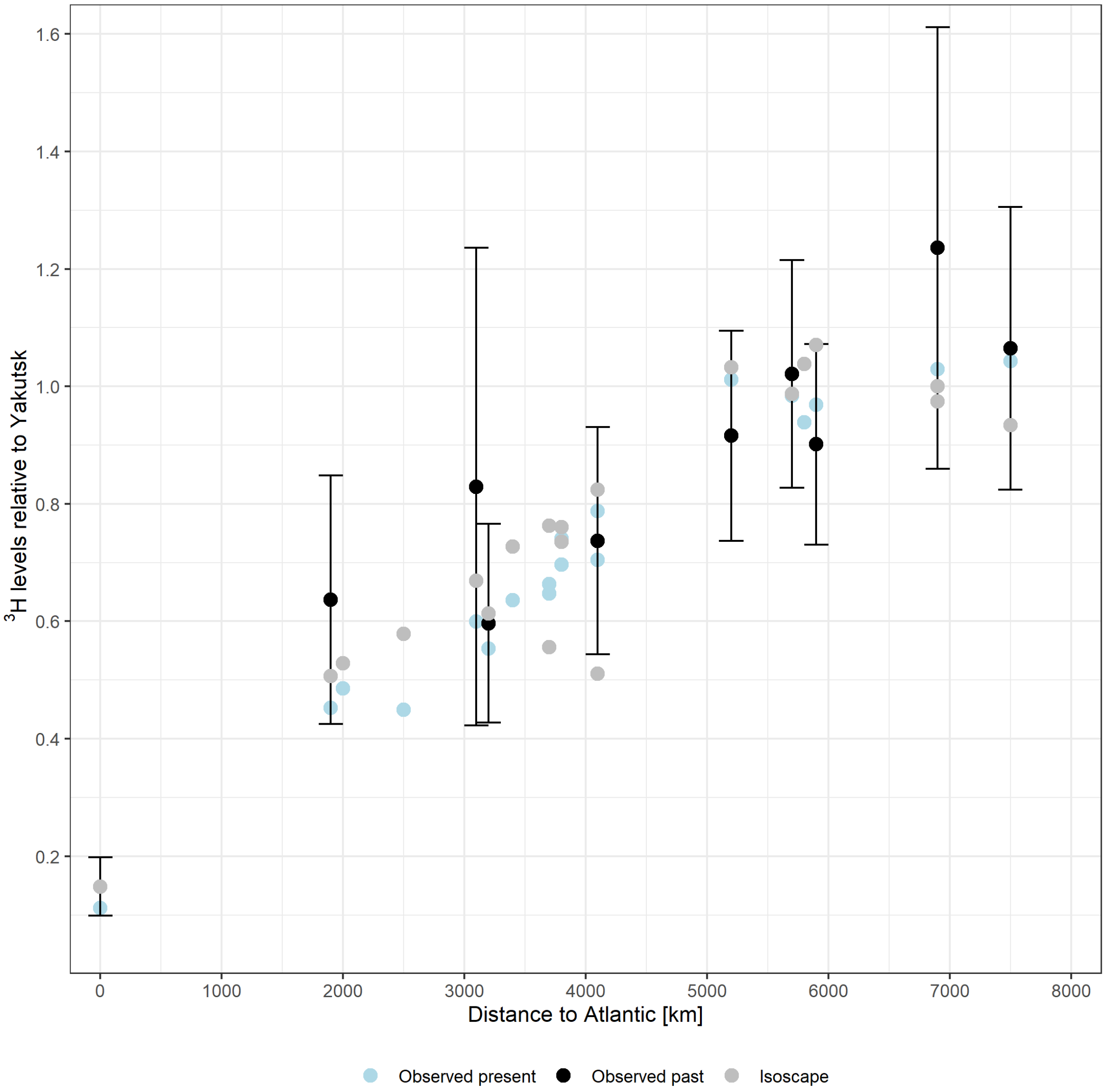

Figure S4.2: Relative ^{3}H levels of several Russian GNIP stations compared to Yakutsk vs. distance to the Atlantic moisture sources using various prediction techniques. See text for further details. Figure created in R 4.1.0 (with ggplot2 3.3.5 library, all https://cran.r-project.org/).

SM5 Prediction outputs

Station	Latitude	Longitude	Altitude	^{3}H obs.	^{3}H pred.	bias	
	[°N]	[°E]	[m.a.s.l.]	[TU]	[TU]	[TU]	[%]
ADELAIDE (AIRPORT)	-34.95	138.52	2	2.6	2.7	0.1	+4.4
ALERT	82.50	-62.33	62	12.2	9.6	-2.6	-21.4
ALICE SPRINGS (AIRPORT)	-23.80	133.89	546	2.6	2.5	-0.1	-2.7
ALMERIA	36.85	-2.38	21	3.5	4.1	0.7	+18.9
ALPA CORRAL	-32.69	-64.72	826	11.2	6.5	-4.7	-41.9
ANADYR	64.73	177.53	35	10.0	10.3	0.4	+3.8
ANCHORAGE (ALASKA)	61.17	-150.02	35	7.8	7.3	-0.5	-6.1
ARATA	-35.64	-64.36	219	7.1	5.9	-1.2	-16.3
ARKHANGELSK	64.58	40.50	13	11.5	12.0	0.5	+3.9
Asahikawa Kaki N	43.77	142.36	11	3.6	5.7	2.1	+58.3
ASCENSION IS.	-7.92	-14.42	15	1.1	1.0	-0.1	-10.0
ASMARA	15.33	38.93	2345	5.5	2.4	-3.1	-56.4
ASTRAKHAN	46.25	48.03	-18	15.0	10.0	-5.0	-33.5
AVIGNON	43.95	4.82	30	8.5	6.3	-2.2	-26.2
AYABACA	-4.63	-79.72	2727	2.0	2.0	0.0	+0.1
BAD LIPPSPRINGE	51.78	8.82	145	8.3	8.3	0.0	+0.2
BAHRAIN	26.27	50.62	2	2.9	3.6	0.7	+24.1
BAKURIANI	41.73	43.52	1665	9.8	9.4	-0.4	-4.3
Baldim	-19.29	-43.96	675	2.0	2.2	0.2	+9.9
BAMAKO	12.69	-8.00	381	4.0	3.8	-0.2	-4.5
BANGKOK	13.73	100.50	2	1.6	1.8	0.2	+13.1
BANKASS	14.08	-3.52	300	4.8	4.8	0.0	+0.1
BARISAL	22.70	90.36	9	3.3	3.5	0.2	+6.6
BELO HORIZONTE	-19.87	-43.97	857	2.5	2.3	-0.2	-7.5
BERLIN	52.47	13.40	50	8.5	9.2	0.6	+7.5
BOBO-DIOULASSO	11.18	-4.30	450	3.4	3.5	0.1	+2.0
BOGOTA	4.70	-74.13	2547	1.9	2.2	0.3	+16.6
BRAUNSCHWEIG	52.30	10.45	88	8.4	8.7	0.3	+3.5
BRISBANE (AERO)	-27.39	153.13	4	1.8	1.6	-0.2	-9.8
Bundaberg	-24.87	152.35	14	1.0	1.5	0.5	+53.5
CACERES	39.47	-6.33	405	3.5	2.9	-0.5	-15.5
Camp Verde	34.55	-111.85	949	6.9	5.3	-1.6	-23.3
CAMPBELLTOWN	-34.40	150.00	115	2.2	2.4	0.2	+11.2
CAPE GRIM (BAPS)	-40.68	144.69	94	2.2	2.3	0.1	+4.4
CAPE TOWN APT.	-33.97	18.60	44	2.1	1.9	-0.2	-9.0
Cave Creek	33.83	-111.93	661	3.6	5.1	1.5	+43.0
CERI Sapporo (Uni)	43.04	141.36	37	3.9	5.5	1.6	+39.8
CHARCHES (VALLE DEL ZALABI)	37.29	-2.96	1426	3.8	4.0	0.2	+5.9
CHARLEVILLE (AERO)	-26.41	146.26	302	2.2	2.1	-0.1	-3.7
CHARLOTTETOWN	46.29	-63.12	49	6.9	6.5	-0.4	-5.1
CHEBOKSARY	56.08	47.33	150	13.4	14.9	1.5	+11.2
Chiba Inage-ku	35.64	140.11	25	2.6	2.0	-0.6	-24.1
CHURCHILL	58.75	-94.06	5	9.8	12.2	2.4	+24.2
CIUDAD REAL	38.98	-3.92	682	3.8	4.0	0.3	+7.4
Claredale	-19.58	147.41	11	1.0	1.4	0.4	+43.4
COTONOU (IITA)	6.42	2.33	14	1.5	1.9	0.4	+25.4
Coutances	49.04	-1.46	95	2.4	6.0	3.6	+152.9
CUXHAVEN	53.87	8.72	12	9.8	8.1	-1.7	-17.5
DAKAR YOFF	14.73	-17.50	24	2.6	2.3	-0.3	-12.8
DANMARKSHAVN	76.77	-18.67	12	7.4	9.6	2.2	+30.2
DARWIN (AIRPORT)	-12.42	130.89	30	1.4	1.2	-0.2	-14.6
DHAKA	23.95	90.28	14	3.9	3.8	-0.1	-3.4
Dieng Geoth	-7.21	109.90	2150	2.7	2.2	-0.5	-19.6
DILIMAN QUEZON CITY	14.64	121.04	42	1.2	1.1	-0.1	-10.1
DISKO ISLAND (QEQERTARSUAQ)	69.27	-53.47	80	9.2	11.5	2.3	+24.7
DOAKTOWN (N.B.)	46.59	-66.01	43	8.0	8.0	0.0	-0.2
DODOMA	-6.19	35.75	1157	1.5	1.2	-0.3	-17.2
DOUALA-HYDRAC	4.04	9.73	18	1.8	1.7	-0.1	-6.9
DUSHANBE	38.55	68.78	1600	9.9	10.8	1.0	+9.7
EMMERICH	51.83	6.60	43	8.7	7.9	-0.7	-8.5
ENISEJSK	58.45	92.15	78	20.2	20.9	0.6	+3.2
ENTEBBE	0.05	32.45	1155	1.7	1.8	0.1	+5.3
ESPOO	60.18	24.83	30	9.3	10.9	1.6	+17.2

FES SAIS	33.97	-4.98	571	2.8	3.6	0.8	+28.5
FLAGSTAFF (ARIZONA)	35.13	-111.67	2137	8.9	7.4	-1.5	-16.6
Freemont	37.58	-122.00	16	4.6	3.7	-0.9	-19.6
FUNCHAL (MADEIRA UNIV.)	32.66	-16.92	213	1.2	1.4	0.2	+17.5
GALAPAGOS	-0.90	-89.61	15	0.5	-0.4	-0.9	-182.2
GARMISCH-P.	47.48	11.07	720	8.9	8.7	-0.2	-2.7
GERONA	41.90	2.75	129	4.1	5.4	1.3	+31.8
GIBRALTAR	36.15	-5.35	5	2.6	3.0	0.3	+13.0
Goa	15.29	74.12	77	3.1	3.0	-0.1	-3.5
GOMA	-1.68	29.23	1535	2.0	1.9	0.0	-2.2
GORODETS	56.68	43.43	90	13.3	14.2	0.9	+6.5
GOUGH IS.	-40.35	-9.88	54	1.9	1.7	-0.1	-8.0
GRIMSEL	46.57	8.33	1950	9.4	8.1	-1.3	-14.1
GRONINGEN	53.23	6.55	1	10.0	7.9	-2.0	-20.6
GUTTANNEN	46.66	8.29	1055	9.1	8.0	-1.1	-12.1
HANOI (IGS)	21.03	105.84	11	2.4	2.2	-0.2	-8.4
HAVANA (CPHR)	23.05	-82.22	137	1.4	0.7	-0.7	-47.5
HEREDIA (UN)	9.93	-84.10	1150	1.2	1.1	-0.1	-4.6
HOF-HOHENSAAS	50.32	11.88	567	8.6	8.7	0.1	+1.5
HONG KONG	22.32	114.17	66	1.9	1.9	0.0	+0.1
HONGSEONG	36.56	126.64	62	4.2	5.3	1.2	+28.0
IRKUTSK	52.27	104.35	485	21.3	19.0	-2.3	-10.7
ISLA DE PASCUA	-27.17	-109.43	41	1.1	-0.7	-1.8	-167.5
ISLAMABAD-NILORE	33.66	73.27	575	4.5	7.5	3.0	+66.3
IZOBAMBA	-0.37	-78.55	3058	2.2	2.0	-0.2	-9.3
KAITOKE	-41.10	175.17	200	1.9	1.9	0.0	+1.4
KALININGRAD	54.70	20.62	5	9.9	10.3	0.4	+4.2
KAMENSKOE	62.47	166.21	30	11.2	10.6	-0.6	-5.2
KARLSRUHE	49.02	8.38	120	8.5	8.1	-0.4	-4.6
KAZAN	55.78	49.13	60	15.8	14.8	-0.9	-6.0
KHABAROVSK	48.52	135.17	72	16.2	12.5	-3.7	-22.7
KHOLMSK	47.05	142.05	29	9.0	9.8	0.8	+8.8
KISANGANI	0.51	25.21	418	2.9	2.8	-0.1	-2.9
KOBLENZ	50.35	7.58	97	8.8	7.8	-1.0	-11.4
KONSTANZ	47.68	9.18	447	9.4	8.6	-0.8	-8.3
KOUCHIBOUGUAC (NB)	46.77	-65.00	21	7.9	7.6	-0.3	-4.1
KRAKOW	50.06	19.85	205	9.3	10.5	1.2	+12.8
KUMAMOTO	32.81	130.73	27	2.3	2.0	-0.4	-16.3
KUOPIO	62.89	27.63	116	9.6	11.3	1.7	+17.3
LA BREVINE	46.98	6.61	1042	8.0	7.6	-0.4	-4.7
LA CORUNA	43.37	-8.42	57	2.4	3.2	0.9	+36.1
LA PERAL	43.04	-6.25	1355	3.6	3.7	0.1	+1.4
Lagoa Santa	-19.63	-43.90	819	2.1	2.3	0.2	+7.4
LEIPZIG	51.35	12.43	125	8.9	8.8	-0.1	-0.8
LEON	42.58	-5.65	913	4.1	3.7	-0.3	-8.5
LEOVA	46.50	28.30	156	11.1	10.1	-1.0	-8.8
LIBREVILLE	0.32	9.44	69	1.3	1.6	0.3	+22.8
LISBON (ITN)	38.79	-9.11	12	1.9	2.1	0.2	+12.6
LJUBLJANA (REAKTOR)	46.10	14.60	282	9.2	8.8	-0.4	-4.4
LOCARNO	46.17	8.79	379	6.5	7.4	0.9	+14.5
Longreach	-23.44	144.28	192	1.8	1.9	0.1	+7.5
Los Angeles	34.07	-118.17	122	2.5	3.4	0.9	+37.2
LOUGA	15.62	-16.22	38	2.5	2.8	0.3	+13.4
Mackay M.O.	-21.12	149.22	30	1.0	1.4	0.4	+40.9
MADRID-RETIRO	40.41	-3.68	655	4.9	4.2	-0.7	-14.0
MAGADAN	59.55	150.78	70	10.2	11.2	1.0	+9.3
MAR DEL PLATA	-38.00	-57.55	10	5.3	3.2	-2.1	-39.2
MARCAPOMACOCHA	-11.40	-76.33	4477	3.4	2.5	-0.9	-27.2
MARGATE (Hobart)	-42.95	147.32	176	2.6	2.9	0.3	+10.3
MARION IS.	-46.88	37.87	26	2.1	2.1	0.0	+1.3
Matsuyama	33.84	132.77	32	3.7	2.0	-1.6	-44.6
MAYAGUEZ	18.21	-67.14	10	1.1	1.0	0.0	-4.2
MECHANIC SETTLEMENT (NB)	45.69	-65.17	403	6.8	6.5	-0.3	-4.2
MEIRINGEN	46.73	8.19	632	9.3	8.0	-1.3	-14.3
MELBOURNE (AIRPORT)	-37.66	144.83	113	3.0	2.7	-0.3	-10.2
MONTEVIDEO-IGS	-34.90	-56.17	37	4.5	2.9	-1.6	-36.0
MORON BASE SEVILLA	37.15	-5.62	88	3.3	2.9	-0.4	-11.6

MOSCOW-BALTSCHUG	55.75	37.63	150	12.3	13.0	0.7	+5.8
MURCIA	38.00	-1.17	62	5.1	4.2	-0.9	-18.5
MURMANSK	68.97	33.05	46	10.1	8.9	-1.2	-11.7
Mutsu	41.29	141.18	12	5.1	4.5	-0.6	-12.1
Nagaoka	37.44	138.94	205	3.0	3.0	-0.1	-1.8
Narrabri	-30.34	149.76	212	2.6	2.8	0.2	+8.0
N'DJAMENA	12.13	15.03	300	5.2	4.9	-0.3	-5.5
Niigata	37.87	138.94	26	4.6	3.5	-1.1	-24.1
NOGUERA DE ALBARRACIN (D.G.A.)	40.46	-1.60	1449	4.1	5.0	0.9	+21.8
NOVOSIBIRSK	55.03	82.90	162	20.1	19.2	-0.8	-4.1
NY ALESUND	78.25	11.92	7	5.6	6.1	0.5	+9.5
NYON	46.40	6.23	436	8.0	7.3	-0.7	-8.3
Oakland	37.84	-122.23	6	1.9	3.7	1.8	+98.6
OGA	39.88	139.85	10	4.0	4.1	0.1	+2.8
OLENEK	68.50	112.43	220	18.6	18.9	0.3	+1.5
OMSK	55.01	73.38	94	20.0	20.1	0.2	+0.8
ORLEANS-LA-SOURCE	47.90	1.90	109	5.3	6.3	1.0	+18.6
OSTROV DIKSON	73.52	80.40	5	13.6	13.7	0.1	+0.7
OTTAWA	45.32	-75.67	114	14.5	9.2	-5.2	-36.3
OUAGADOUGOU	12.38	-1.50	298	3.7	4.0	0.4	+9.6
PALMA DE MALLORCA	39.55	2.62	3	3.9	4.0	0.1	+3.0
PERM	58.01	56.18	161	16.6	16.1	-0.5	-3.0
PERTH (AIRPORT)	-31.93	115.98	18	1.8	1.9	0.1	+3.6
PETROPAVLOVSK-KAMCHATSKIY	52.98	158.65	24	7.1	7.0	-0.1	-1.1
PEVEK	69.70	170.25	20	12.8	12.9	0.1	+0.5
PONTA DELGADA (AZORES)	37.77	-25.65	175	1.8	0.3	-1.5	-83.9
PONTRESINA	46.49	9.90	1724	7.7	8.3	0.7	+8.6
PORTO	41.13	-8.60	93	2.3	2.7	0.4	+17.5
POUT	14.77	-17.06	19	2.2	2.3	0.1	+4.0
PRAGUE/WRI	50.12	14.39	184	9.5	9.5	-0.1	-0.7
PUERTO ALMENDRAS - IQUITOS	-3.82	-73.38	98	2.0	2.6	0.5	+26.3
PUERTO DE NAVACERRADA	40.79	-4.01	1894	3.7	4.2	0.5	+12.8
PUERTO MONTT	-41.47	-72.93	13	2.0	3.7	1.8	+89.0
Puncak Jaya glacier	-4.84	137.18	4800	2.0	2.3	0.3	+13.5
RAMNICU VALCEA	45.04	24.28	237	10.7	9.3	-1.4	-13.5
REGENSBURG	49.03	12.12	377	8.7	8.9	0.2	+2.8
REUNION	-20.90	55.48	70	1.0	1.0	0.0	+2.5
REYKJAVIK	64.13	-21.93	14	3.8	4.0	0.2	+4.9
RIO CLARO (UNESP)	-22.40	-47.54	614	2.4	2.8	0.4	+18.0
RIO CUARTO	-33.11	-64.25	435	6.5	6.9	0.4	+6.8
RIYADH	24.72	46.64	613	3.8	3.3	-0.5	-12.6
ROTHERA POINT	-67.57	-68.13	5	4.0	3.7	-0.3	-7.8
ROVANIEMI	66.50	25.76	107	9.8	9.7	0.0	-0.3
Rydee	-33.85	151.10	15	2.2	2.7	0.5	+20.9
SALEKHARD	66.53	66.67	16	15.9	14.5	-1.5	-9.2
SALLENT DE GALLEGO - LA SARRA	42.79	-0.33	1460	4.0	5.5	1.5	+37.1
SALTA	-24.78	-65.40	1187	3.7	5.5	1.9	+51.0
SANTA CRUZ DE TENERIFE	28.45	-16.25	36	1.7	1.8	0.1	+5.8
SANTA ROSA	-36.62	-64.60	180	7.1	5.6	-1.6	-22.2
SANTANDER	43.48	-3.80	52	3.2	3.8	0.6	+20.3
SANTIAGO	-33.45	-70.70	520	2.6	3.9	1.3	+48.6
SANTIAGO DEL ESTERO	-27.78	-64.27	187	4.7	7.1	2.3	+48.9
SANTO DOMINGO	18.48	-69.92	14	1.1	1.1	0.0	-1.5
Sapporo	43.07	141.35	25	6.0	5.5	-0.6	-9.4
SFAX	34.72	10.68	23	4.1	4.2	0.1	+2.4
SFS Prioksko-Terrasny Reserve	54.90	37.55	166	12.0	13.1	1.1	+9.3
Sierra Nevada	39.27	-120.11	1775	4.4	4.2	-0.2	-5.5
SION	46.22	7.34	482	7.9	7.6	-0.3	-3.8
SITIO DAS FONTES	37.01	-7.96	9	1.2	2.1	0.9	+81.4
SKOVORODINO	54.00	123.97	400	21.3	18.2	-3.1	-14.4
Southern Sierra Critical Zone Observatory	37.06	-119.21	2000	3.9	3.6	-0.3	-7.5
ST. GALLEN	47.43	9.40	779	9.6	8.4	-1.1	-11.8
Stadviliai	55.73	26.18	150	10.0	11.0	1.0	+9.7
STUTTGART	48.83	9.20	315	8.2	8.3	0.1	+1.0
SYLHET	24.91	91.85	20	3.2	3.2	0.0	+0.2
TBILISI	41.75	44.77	427	11.1	8.3	-2.8	-25.4
TELAVI	41.93	45.48	568	10.1	9.1	-1.0	-10.3

TETYUSHI	54.95	48.78	160	14.7	14.3	-0.4	-2.6
THESSALONIKI (UNIV.)	40.63	22.96	200	6.9	6.2	-0.6	-9.3
THONON-LES-BAINS	46.37	6.47	385	9.3	7.5	-1.8	-19.2
TIKSI	71.58	128.92	10	15.8	14.8	-1.0	-6.5
Toki	35.32	137.17	260	1.7	2.5	0.9	+51.5
Toowoomba	-27.58	151.93	691	1.8	2.4	0.6	+31.5
TORTOSA	40.81	0.52	48	5.0	4.8	-0.2	-4.5
Toulumme	37.88	-119.36	795	4.6	5.3	0.7	+15.9
TRAVELLER'S HILL	-7.94	-14.37	400	0.8	1.0	0.2	+24.0
TRIER	49.75	6.70	273	8.5	7.5	-1.0	-11.8
TSIMLYANSK	47.63	42.12	70	13.3	10.8	-2.5	-18.5
TSUKUBA (UNIVERSITY)	36.11	140.10	27	2.3	2.3	0.0	+2.0
TUCSON AZ	32.24	-110.94	753	5.3	4.8	-0.5	-9.8
TUCUMAN	-26.82	-65.22	430	5.1	6.0	0.9	+17.6
TULENAPA	7.77	-76.67	30	1.2	0.9	-0.3	-25.0
TURA	64.33	100.43	209	18.8	20.2	1.4	+7.7
UHLIRSKA	50.83	15.15	823	8.6	9.5	1.0	+11.2
ULAANBAATAR	47.93	106.98	1338	26.5	15.8	-10.7	-40.4
VALENCIA	39.47	-0.38	13	5.8	4.2	-1.6	-27.0
VALENTIA (OBSERVATORY)	51.93	-10.25	9	2.6	2.9	0.3	+10.8
VALLADOLID	41.63	-4.77	735	4.3	3.9	-0.4	-8.8
VANCOUVER	49.19	-123.02	4	4.4	7.0	2.6	+58.2
VERHOJANSK	67.57	133.40	127	16.8	17.2	0.4	+2.3
VERNADSKY (ARGENTINE IS.)	-65.08	-63.98	20	3.3	3.4	0.1	+2.7
VIENNA (HOHE WARTE)	48.25	16.36	198	9.1	9.9	0.8	+9.0
WASSERKUPPE RHOEN	50.50	9.95	921	8.5	8.4	-0.1	-1.7
WEIL AM RHEIN	47.60	7.59	249	8.7	8.2	-0.6	-6.4
WHITEHORSE	60.72	-135.07	702	8.2	8.5	0.3	+4.3
WUERZBURG	49.80	9.90	259	9.0	8.3	-0.7	-7.5
XALAPA	19.53	-96.91	1390	1.8	1.7	-0.1	-6.7
YAKUTSK	62.08	129.75	107	20.8	19.5	-1.3	-6.4
ZAGREB-GRIC	45.82	15.98	157	8.1	8.9	0.7	+8.8
ZARAGOZA	41.67	-1.02	247	4.8	5.0	0.1	+2.6
Zittau	50.90	14.80	240	8.3	9.5	1.2	+15.0

Table S5: Observed vs. predicted 3H levels for the observed data points. Absolute bias in red indicates that the predicted value is ≥ the observed plus assigned isoscape uncertainty (0.9 TU). Green indicates a prediction result ≤ observed minus uncertainty.

References

[1] Palcsu, L., Morgenstern, U., Sültenfuss, J., Koltai, G., László, E., Temovski, M., Major, Z., Nagy, J.T., Papp, L., Varlam C., Faurescu, I., Túri, M., Rinyu, L., Czuppon, G., Bottyán, E. and Jull, A.T.J., 2018. Modulation of Cosmogenic Tritium in Meteoric Precipitation by the 11-year Cycle of Solar Magnetic Field Activity. *Scientific Reports* **8**, 12813.

[2] Morgenstern, U., and Taylor, C.B., 2009. Ultra low-level tritium measurement using electrolytic enrichment and LSC. *Isotopes in Environmental and Health Studies* **45 (2)**, 96-117.

[3] Ansari, M.A., Mohokar, H.V., Deodhar, A., Jacob, N. and Sinha, U.K., 2018. Distribution of environmental tritium in rivers, groundwater, mine water and precipitation in Goa, India. *Journal of environmental radioactivity*, **189**, 120-126.

[4] Bolzan, F. et al., 2019. "Disappearance of the last tropical glaciers in the Western Pacific Warm Pool (Papua, Indonesia) appears imminent." *Proceedings of the National Academy of Sciences* **116 (52)**, 26382-26388.

[5] Connan, O., Maire, D., Hébert, D., Solier, L., Laguionie, P., Rozet, M., Lamotte, M. and Maro, D., 2020. Tritium in precipitation on 5 sites in North-West France during the 2017–2019 period. *Journal of Environmental Radioactivity*, **212**, 106129.

[6] Eastoe, C.J., Watts, C.J., Ploughe, M. and Wright, W.E., 2012. Future use of tritium in mapping pre-bomb groundwater volumes. *Groundwater*, **50(1)**, 87-93.

[7] Gusyev, M. A., U. Morgenstern, T. Nishihara, T. Hayashi, N. Akata, K. Ichiyanagi, A. Sugimoto, A. Hasegawa, and M. K. Stewart. 2019 "Evaluating anthropogenic and environmental tritium effects using precipitation and Hokkaido snowpack at selected coastal locations in Asia." *Science of The Total Environment* **659**, 1307-1321.

[8] Harms, P.A., Visser, A., Moran, J.E. and Esser, B.K., 2016. Distribution of tritium in precipitation and surface water in California. *Journal of Hydrology*, **534**, 63-72.

[9] International Atomic Energy Agency, 2020. Global Network of Isotopes in Precipitation. The GNIP Database. Vienna: International Atomic Energy Agency. Retrieved from https://nucleus.iaea.org/wiser (accessed on 10 October 2019).

[10] Jefanova, O., Mažeika, J., Petrošius, R., Skuratovič, Ž., Paškauskas, R., Martma, T., Liblik, T. and Ezhova, E., 2020. Baltic Sea water tritium and stable isotopes in 2016–2017. Isotopes in *Environmental and Health Studies*, **56(2)**, 193-204.

[11] Michelsen, N., Reshid, M., Siebert, C., Schulz, S., Knöller, K., Weise, S.M., Rausch, R., Al-Saud, M. and Schüth, C., 2015. Isotopic and chemical composition of precipitation in Riyadh, Saudi Arabia. *Chemical Geology*, **413**, 51-62.

[12] Permana, D. S. 2011 "Climate, precipitation isotopic composition and tropical ice core analysis of Papua, Indonesia." PhD diss., The Ohio State University.

[13] Ribeiro, C., Velásquez, L., and Fleming, P. 2020 "Origin of spring waters employing a multiparametric approach with special focus on stable isotopes ^{2}H and ^{18}O in the Lagoa Santa Karst region, Southern Brazil." *Isotopes in Environmental and Health Studies*, **56(2)**, 158-169.

[14] RPA Typhoon, 2016. Radiation Situation in the Russian Territory and Neighbouring States 2016 (Yearbook), https://www.rpatyphoon.ru/upload/medialibrary/130/ezhegodnik_ro_2016.pdf

[15] RPA Typhoon, 2017. Radiation Situation in the Russian Territory and Neighbouring States 2016 (Yearbook), https://www.rpatyphoon.ru/upload/medialibrary/1c9/ezhegodnik_ro_2017.pdf

[16] RPA Typhoon, 2018. Radiation Situation in the Russian Territory and Neighbouring States 2016 (Yearbook), https://www.rpatyphoon.ru/upload/medialibrary/653/ezhegodnik_ro_2018.pdf

[17] Tadros, C.V., Hughes, C.E., Crawford, J., Hollins, S.E. and Chisari, R., 2014. Tritium in Australian precipitation: A 50 year record. *Journal of hydrology*, **513**, 262-273.

[18] Visser, A., Thaw, M. and Esser, B., 2018. Analysis of air mass trajectories to explain observed variability of tritium in precipitation at the Southern Sierra Critical Zone Observatory, California, USA. *Journal of environmental radioactivity*, **181**, 42-51.

[19] Webster-Brown, J., Gall, M., Gibson, J., Wood, S. and Hawes, I., 2010. The biogeochemistry of meltwater habitats in the Darwin Glacier region (80 S), Victoria Land, Antarctica. *Antarctic Science*, **22(6)**, 646-661.

[20] Kotzer, T.G., Kudo, A., Zheng, J. and Workman, W., 2000. Natural and anthropogenic levels of tritium in a Canadian Arctic ice core, Agassiz Ice Cap, Ellesmere Island, and comparison with other radionuclides. *Journal of Glaciology*, **46(152)**, 35-40.

[21] Roether, W. 1967. Estimating the tritium input to groundwater from wine samples: Groundwater and direct run-off contribution to Central European surface waters. International Atomic Energy Agency (IAEA): *Applications of Isotopes in Hydrology*, 73-91

[22] Kaufman, S. and Libby, W.F., 1954. The natural distribution of tritium. *Physical Review*, **93(6)**, 1337.

[23] Verhagen, B.T. and Nichola, A., 1982. Isotopes. Veritas in Vino. *Nucl. Act*. **27**, 18-21.

[24] Von Buttlar, H. and Libby, W.F., 1955. Natural distribution of cosmic-ray produced tritium. II. *Journal of Inorganic and Nuclear Chemistry*, **1(1-2)**, 75-91.

[25] Allison, G.B. and Hughes, M.W., 1977. The history of tritium fallout in southern Australia as inferred from rainfall and wine samples. *Earth and Planetary Science Letters*, **36(2)**, 334-340.
